# Fecal microbiota transplantation alleviates lipopolysaccharide-induced osteoporosis by modulating gut microbiota and long non-coding RNA *TUG1* expression

**DOI:** 10.3389/fcimb.2025.1535666

**Published:** 2025-04-11

**Authors:** Pengcheng Ma, Ruoyi Wang, Huizhi Chen, Jiachun Zheng, Weijie Yang, Bo Meng, Yifan Liu, Yao Lu, Jing Zhao, Hongwei Gao

**Affiliations:** ^1^ Shandong Public Health Clinical Center, Shandong University, Jinan, China; ^2^ Shandong Provincial Key Laboratory of Animal Cells and Developmental Biology, School of Life Sciences, Shandong University, Qingdao, China; ^3^ School of Mechanical Engineering, Shandong University, Jinan, China

**Keywords:** fecal microbiota transplantation, osteoporosis, gut microbiota, lncRNA, lipopolysaccharides

## Abstract

**Purpose:**

To study whether fecal microbiota transplantation (FMT) can alleviate lipopolysaccharide (LPS)-induced osteoporosis (OP) by regulating the composition and abundance of gut microbiota and the expression level of long non-coding RNA (lncRNA) *TUG1*.

**Methods:**

Twenty C57BL/6 mice were selected. Two mice were randomly designated as fecal donors, while the remaining mice were randomly divided into control group, LPS group, and LPS + FMT group. Each group consisted of 6 mice. The mice in the LPS and LPS + FMT groups were intraperitoneally injected with LPS to establish the OP model, and the mice in the LPS + FMT group were treated with donor feces by gavage. Micro-CT was used to scan the femur specimens of mice, and the bone structural parameters of the control and LPS groups were compared to verify the effectiveness of the OP model. HE staining was used to compare the microstructure of femurs in the 3 groups. 16S rRNA gene sequencing was used to analyze the composition and abundance of gut microbiota in mice. Immunofluorescence staining was used to compare the expression levels of Runt-related transcription factor 2 (RUNX2) in the femur of the 3 groups. Real-time quantitative reverse transcription PCR (qRT-PCR) was used to compare the expression levels of lncRNA *TUG1* in the intestines and serum of mice in the 3 groups.

**Results:**

Micro-CT showed that compared with the control group, the mice in the LPS group had more bone loss. The bone mineral density, trabecular number, and trabecular thickness of the control group was higher, and the trabecular separation was smaller. The models were validated effectively. HE staining showed that compared with the control group, the bone trabeculae in the LPS group were thinner and sparse, while that in the LPS + FMT group were dense and clear. The 16s rRNA sequencing showed that the abundance of Bacteroides and Lactobacillus in LPS+FMT group was significantly higher than that in LPS group. Immunofluorescence staining showed that the RUNX2 level in the control group and LPS + FMT group was similar, and both were higher than that in the LPS group. The qRT-PCR results showed that the *TUG1* mRNA level in the control group and LPS + FMT group was similar and significantly higher than that in the LPS group.

**Conclusion:**

FMT can enhance osteoblast levels and improve bone structure by modulating the abundance of gut microbiota in OP mice (such as increasing Bacteroides and Lactobacillus populations) and promoting the expression of lncRNA *TUG1*, thereby alleviating LPS-induced OP.

## 1 Introduction

Osteoporosis (OP) is a metabolic disease characterized by osteopenia and destruction of bone microstructure, which can increase the risk of fracture. The pain and deformity caused by OP seriously reduce the quality of patients’ survival (Anish and Nair, 2024). More than 150 million people are diagnosed with OP every year, and the high incidence has made it a global public health problem that needs to be solved urgently (Zhang et al., 2021). The pathogenesis of OP is imbalance of bone metabolism, and drugs to restore the homeostasis of bone formation and resorption are the main means of treating OP. However, long-term use of anti-OP drugs may cause side effects such as muscle pain and renal insufficiency, resulting in poor patient compliance (Reid and Billington, 2022). Currently, finding safer and more effective strategies for the prevention and treatment of OP has become a research hotspot.

Gut microbiota is the microbial community inhabiting the human gastrointestinal tract, which plays an important role in regulating bone development and biomechanics of bone tissue (Guss et al., 2017; Orwoll et al., 2022). Gut microbiota can delay the progression of OP by regulating the immune system and reducing the production of tumor necrosis factor. Gut microbiota can also indirectly regulate bone homeostasis through endocrine bone signaling factors such as estrogen and parathyroid hormone. In addition, metabolites of gut microbiota, such as short-chain fatty acids and 5-hydroxytryptamine, can affect the number and activity of osteoblasts and osteoclasts (Hao et al., 2024). The phylum of gut microbiota includes Firmicutes, Bacteroidetes, etc., and bacteria from different phyla have different effects on OP (Marchesi et al., 2016). It has been noted that bone mass loss was significantly improved in OP patients after oral administration of Lactobacillus (Li et al., 2022). Another study suggested that Enterobacteriales could aggravate OP by regulating amino acid metabolism (Wang S. et al., 2022). Therefore, it is necessary to explore new targets in the complex mechanisms by which the gut microbiota affects OP.

The composition and abundance of gut microbiota are affected by many factors. Such as long-term use of antibiotics can induce dysbiosis of gut microbiota and decrease the bending strength of bone. Consumption of certain prebiotics is beneficial for increasing the population of beneficial bacteria in the gut, enhancing the secretion of short-chain fatty acids (SCFAs), and improving the content and density of minerals in bone (Cronin et al., 2016). Fecal microbiota transplantation (FMT) represents a novel “organ transplant” technique that can reestablish a normal gut microbial ecology. Through various mechanisms such as correcting microbial dysbiosis, repairing gut barrier functions, and modulating immune responses, FMT participates in the regulation of bone metabolism (Zhang et al., 2022b). Although clinical evidence is still lacking to prove that FMT can be used to treat patients with OP, animal studies have confirmed the efficacy of FMT in treating OP. Ma’s study demonstrated that transplanting feces from young rats to elderly OP rats can alter their gut microbiota composition, improve gut barrier functions, and ultimately reduce bone loss to alleviate OP in elderly rats (Ma et al., 2021). However, it remains to be investigated whether this phenomenon applies to lipopolysaccharide (LPS)-induced OP, and how the species and quantity of gut microbiota change in LPS-induced OP animal models treated with FMT.

Long non-coding RNA (lncRNA) is a kind of RNA that is not involved in protein coding and has the ability to regulate a variety of physiological and pathological processes (Jathar et al., 2017). For example, lncRNA *TUG1* can promote bone formation through various ways, including binding to Lin28a to promote the osteogenic differentiation of periodontal stem cells, and acting on the Wnt/β-catenin signaling pathway to enhance the proliferation of osteoblasts (He et al., 2018; Liu et al., 2019). The level of lncRNA *TUG1* is regulated by a variety of factors, such as the expression of lncRNA *TUG1* can be affected by the content of taurine, which is a metabolite of some gut bacteria (Young et al., 2005). Therefore, whether FMT can regulate OP by affecting the expression of lncRNA *TUG1* remains to be determined. The aim of this study is to establish the mice model of LPS-induced OP and reshape the gut microbiota by FMT. The changes of gut microbiota and the level of lncRNA *TUG1* were observed to explore whether FMT could alleviate LPS-induced OP by regulating the gut microbiota and the expression of lncRNA *TUG1*, and provide a basis for the biological treatment of OP.

## 2 Materials and methods

### 2.1 Selection and handling of experimental animals

The study followed the principles for the use of experimental animals in the Animal Center of Shandong University and was approved by the Ethics Committee of Shandong University. Twenty 6-week-old C57BL/6 mice with similar body weights were selected. The experimental animals were all of the same strain of healthy mice, free from any diseases, including gastrointestinal ailments, ensuring a healthy digestive system function and a good gut microbial ecology. Two mice were randomly selected as fecal donors, with their gut microbial health was verified. The remaining mice were randomly divided into control, LPS, and LPS+FMT groups, with 6 mice in each group. Since estrogen levels can affect bone metabolism by inhibiting Th17 cell differentiation through estrogen receptor pathways and reducing the production of tumor necrosis factor-β receptor activators, thereby suppressing osteoclast function, and estrogen deficiency can trigger CD4+ T-cell mediated immune responses, producing pre-osteoclast factors, activating osteoclasts, and enhancing bone resorption (Jia et al., 2021; Yu et al., 2021). To minimize the impact of sex hormone level differences on the study outcomes and to avoid bias from using a single gender, the gender ratio in each group was set at 1:1.

Due to the lack of standardized guidelines for FMT, this study draws on the comprehensive review by Bokoliya et al. regarding the operational procedures and precautions for mouse FMT. We followed the FMT principles covered in the review, including, but not limited to, timing fecal collection based on the circadian rhythms of the gut microbiota, establishing the environmental conditions required for fecal processing and storage, and determining the appropriate routes, doses, and frequencies for FMT. Established practices from previous research provide guidance for this study (Bokoliya et al., 2021). All mice were housed in a clean environment with a temperature of (22 ± 1) °C and humidity of (50 ± 5) %, with free access to food and water, under a 12-hour light/dark cycle. Mice in both the LPS and LPS + FMT groups received an intraperitoneal injection of LPS solution at a dose of 10 mg/kg to establish OP models. In contrast, the control group received an equal volume of saline. Injections were administered every other day for a total duration of 10 days (Wang et al., 2020). After this period, fresh feces from the donor mice were collected under sterile conditions. The feces (100 mg) were dissolved in 1 mL of sterile saline, vortexed for 10 seconds, and centrifuged at 2000g for 10 minutes at 4°C to remove any residues. The supernatant was diluted and administered via gavage to the mice in the LPS + FMT group (200 μL/mouse), while the other 2 groups received saline via gavage for 7 days (Yang et al., 2018). Following this treatment, feces and venous blood samples from the mice were collected for further analysis. Subsequently, the mice were euthanized by cervical dislocation, and their right femurs and intestinal tissues were preserved.

### 2.2 Validation of mouse OP model

The femurs were immersed in a 4% paraformaldehyde solution for 24 hours and then scanned using micro-CT with a resolution of 10 μm to create 3-dimensional (3D) reconstructed images of the distal femur. The region between 2% and 7% proximal to the growth plate was selected as the region of interest. Bone mineral density (BMD), trabecular number (Tb. N), trabecular thickness (Tb. Th), and trabecular separation (Tb. Sp) were recorded. The effectiveness of the OP model was validated by comparing CT images and bone structural parameters between the control and LPS groups.

### 2.3 HE staining of mouse femur sections

The decalcified femurs were embedded in paraffin and sectioned. The sections were immersed in hematoxylin staining solution for 3 minutes, rinsed with water, then differentiated in 1% hydrochloric acid alcohol for several seconds before being rinsed again. After the water returned to blue for 3 minutes, the sections were stained with eosin for 3 minutes and rinsed with water again. The sections were dehydrated with gradient ethanol and transparent with xylene, then sealed with neutral resin. The bone tissue staining was observed and compared among the 3 groups.

### 2.4 Analysis of gut microbiota composition and abundance

DNA was extracted from mouse feces using the QIAamp DNA Kit. The bacterial 16S rRNA V3-V4 region was amplified through polymerase chain reaction (PCR) using the forward primer 338F (5’-ACTCCTACGGGAGGCAGCA-3’) and the reverse primer 806R (5’-GGACTACHVGGGTWTCTAAT-3’). The amplification conditions were as follows: a predenaturation at 95°C for 3 minutes, followed by 27 cycles of denaturation at 95°C for 10 seconds, annealing at 55°C for 30 seconds, and extension at 72°C for 30 seconds, stably extension at 72°C for 10 minutes. The samples were stored at 4°C. The amplified products were isolated, and purified in a purification Kit. Quantification of the products was performed using the Quant-iT PicoGreen dsDNA Assay Kit.

Equal concentrations of purified quantitative DNA were mixed to construct a library, which was sequenced using the Illumina HiSeq platform. The gut microbiota data were analyzed using QIIME (v2.0.0), creating bar plots of species distribution at the phylum, class, order, family, and genus levels. The α diversity analysis was conducted using Shannon index, Simpson index, and Chao1 index. Additionally, principal coordinates analysis (PCoA) and non-metric multidimensional scaling (NMDS) were performed. The Chao1 index measures microbial abundance, estimating the total number of microbial taxa in a sample. Both the Shannon and Simpson indices assess microbial diversity within the samples and are positively correlated with diversity. The difference lies in that the Shannon index focuses on quantifying the number of microbial species, whereas the Simpson index emphasizes the evenness of species distribution. PCoA and NMDS are different methods for analyzing β diversity; in PCoA plots, the greater the distance between samples, the greater the intergroup differences in microbial composition. NMDS focuses on reflecting the differences in ranking sample distances between groups, with greater distances indicating larger differences.

### 2.5 Immunofluorescence staining of mouse femur sections

Femur sections were blocked with a 5% BSA solution at room temperature for 30 minutes. The primary antibody (dilution ratio of 1:100) was added and incubated overnight at 4°C. After recovering the primary antibody, the sections were washed 3 times with 0.1M PBS buffer for 5 minutes each. A labeled fluorescent secondary antibody (dilution ratio of 1:200) was then added and incubated in the dark at room temperature for 60 minutes. Following this step, the secondary antibody was discarded, and the sections were washed 3 times with PBS buffer for 10 minutes each. DAPI was added to stain the nuclei in the dark for 10 minutes, followed by 3 washes with PBS for 5 minutes each. Finally, neutral resin was used to mount the sections. The levels of Runt-related transcription factor 2 (RUNX2), a specific marker of type I osteoblasts, were compared among the 3 groups.

### 2.6 Measurement of lncRNA *TUG1* levels in mouse gut and blood

Mouse intestinal tissue was ground into a powder in liquid nitrogen. For every 100 mg of powder, 1 mL of Trizol was added, mixed thoroughly, and allowed to stand for 10 minutes. Mouse venous blood was collected, allowed to stand at 4°C for 30 minutes, then centrifuged at room temperature at 3000 rpm for 15 minutes, and the upper layer of serum was left. Trizol was added to this serum and mixed before allowing it to stand for another 5 minutes.

Chloroform equal to 1/5 of the Trizol volume was added, shaken for 15 seconds, and left at room temperature for an additional 10 minutes. The mixture was then centrifuged at 4°C at 13000 rpm for 10 minutes; isopropanol equal to the supernatant volume was added after discarding the lower phase. Following another centrifugation step at 4°C at 12000 rpm for 10 minutes, the supernatant was discarded. Each tube received 75% ethanol to wash the precipitate before centrifuging again under similar conditions. The precipitate was dried at room temperature for 10 minutes before being dissolved in 30 μL DEPC water. RNA concentration was measured using a micro-spectrophotometer and stored at -80°C.

For reverse transcription, a reaction system of 20 μL was prepared on ice: containing 4 μL of 5×FastKing-RT SuperMix, total RNA (2 μg), and RNase-Free ddH2O to reach a total volume of 20 μL. This mixture was centrifuged after thorough mixing and subjected to a reverse transcription program: incubating at 42°C for 15 minutes followed by heating to 95°C for 3 minutes; cDNA was stored at -20°C.

A real-time quantitative reverse transcription PCR (qRT-PCR) reaction system of 15 μL was prepared: comprising 7.5 μL SYBR Green premix, cDNA template (1.5 μL), forward and reverse primers (0.75 μL each), and ddH2O (4.5 μL). Reaction conditions included predenaturation at 95°C for 10 minutes; denaturation at 95°C for 10 seconds; annealing at 60°C for 30 seconds; extension at 72°C for 30 seconds; followed by termination after completing a total of 40 cycles. Relative expression levels of lncRNA *TUG1* were calculated using the 2^−ΔΔCT^.

### 2.7 Statistical methods

Each experimental procedure was repeated 3 times to obtain results analyzed using SPSS version 26.0. The Shapiro-Wilk test assessed whether experimental data followed a normal distribution; Brown-Forsythe test evaluated homogeneity of variances. Normally distributed data with equal variances were expressed as mean ± standard deviation ( $\bar{x}\pm S$ ), with independent samples *t*-test was used for inter-group comparison, and paired *t*-test was used for intra-group comparison. Graphs were generated using GraphPad Prism version 8 software. *P-*value <0.05 (*) and <0.01 (**) were considered statistically significant.

## 3 Results

### 3.1 Validation of mouse OP model

**Figure 1:**
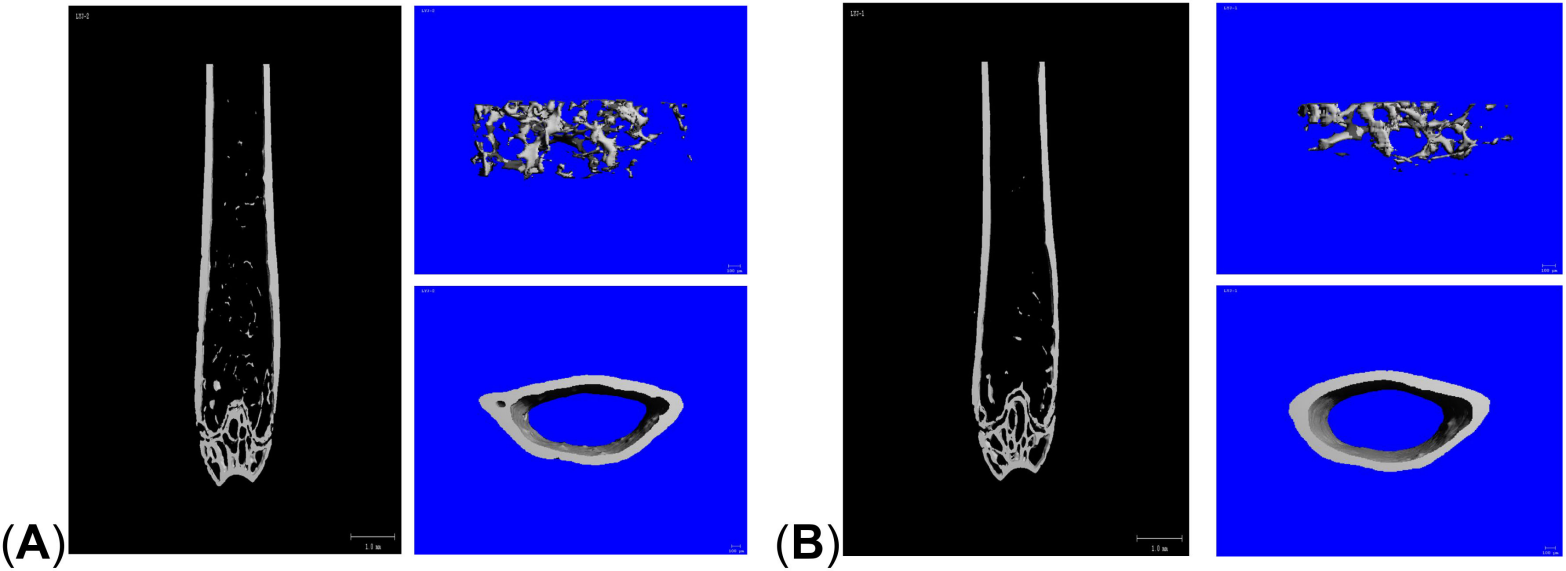
Micro-CT reconstructed images of the distal femur. **(A)** control group; **(B)** LPS group.

**Figure 2:**
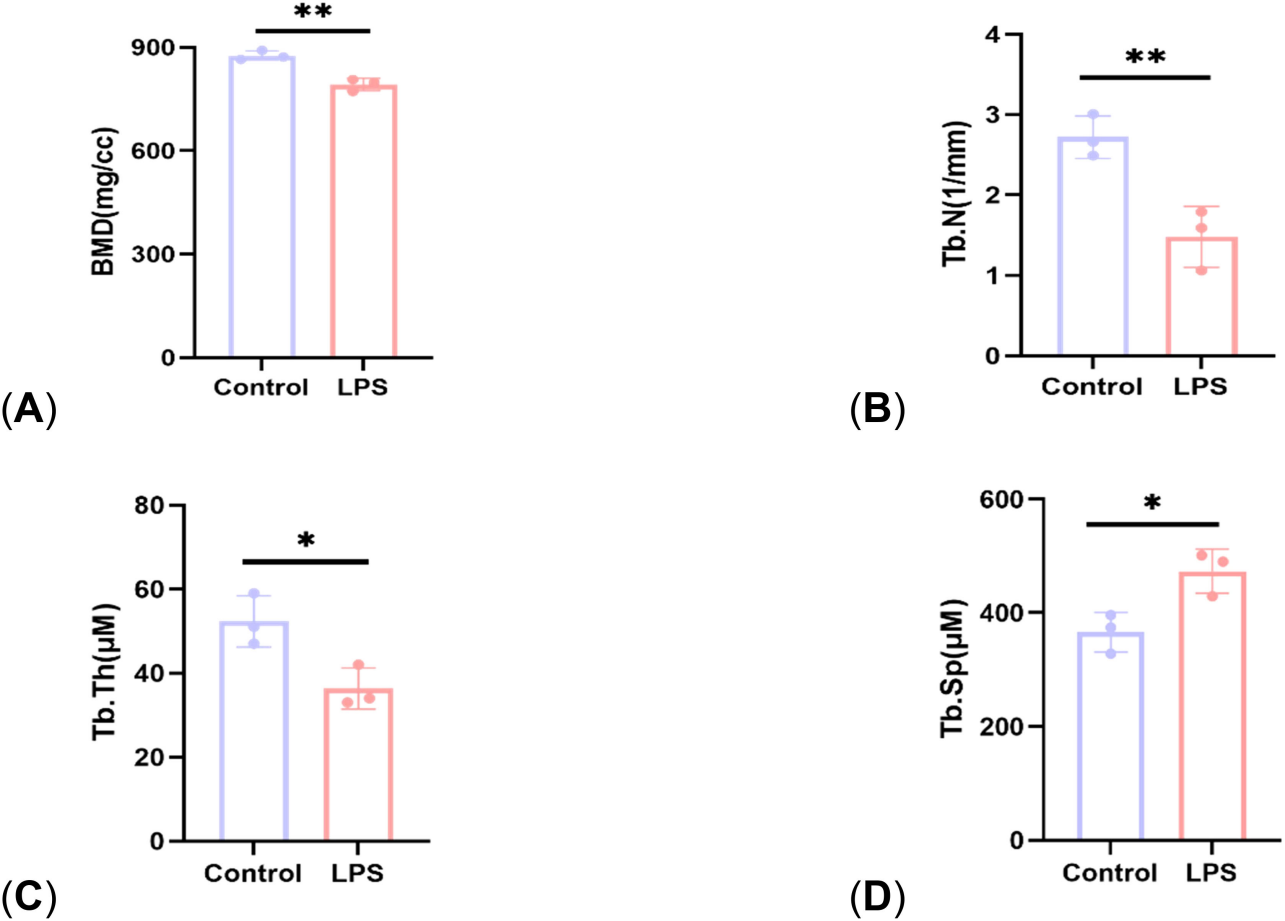
Comparison of bone structural parameters between control group and LPS group. **(A)** BMD; **(B)** Tb. N; **(C)** Tb. Th; **(D)** Tb. Sp. P<0.05 (*), P<0.01 (**).

Micro-CT results showed that compared with the control group, the mice in the LPS group had reduced cancellous bone and thinner cortical bone in the femur (**Figure 1**). The analysis of bone structural parameters showed that the BMD, Tb. N, and Tb. Th of the femur of the mice in the LPS group were smaller than those of the control group, while Tb. Sp was larger than that of the control group, and the differences were all statistically significant (**Figure 2**). The validation results showed that the mouse OP models established in this study were effective.

### 3.2 Comparative of HE staining of femoral sections

**Figure 3:**
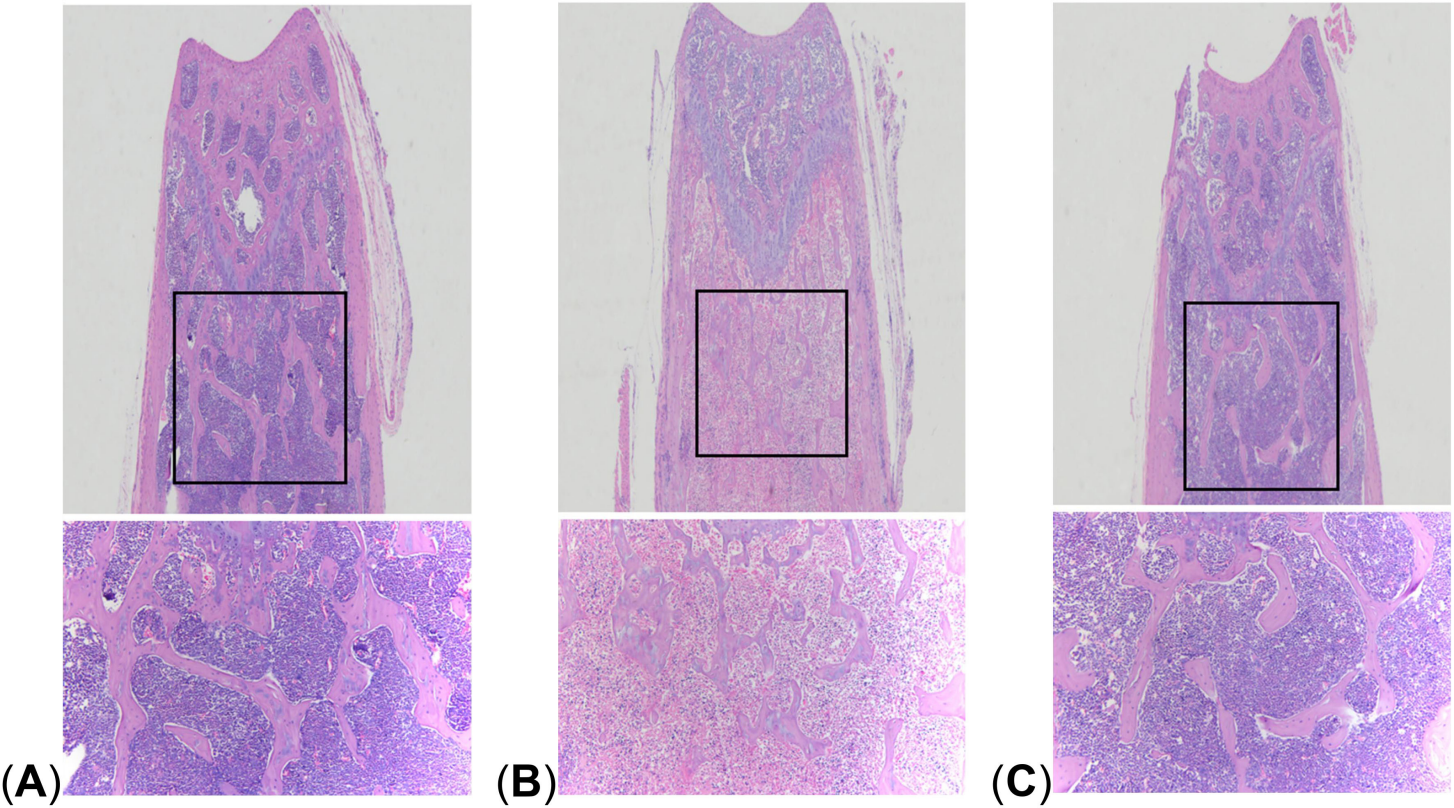
Comparison of HE staining. **(A)** control group; **(B)** LPS group; **(C)** LPS + FMT group.

The results of HE staining showed that compared with the control group, the bone trabeculae in the LPS group were significantly thinner and more widely spaced, and there were large areas of trabecular bone loss. The density of adipocytes increased, showing the pathological characteristics of OP. However, in the LPS + FMT group, the thinning and sparsity of bone trabeculation was significantly improved, and the bone tissue structure was clear, which was basically restored to the same level as the control group, indicating that FMT could alleviate LPS-induced OP (**Figure 3**).

### 3.3 Analysis of gut microbiota composition and abundance

**Figure 4:**
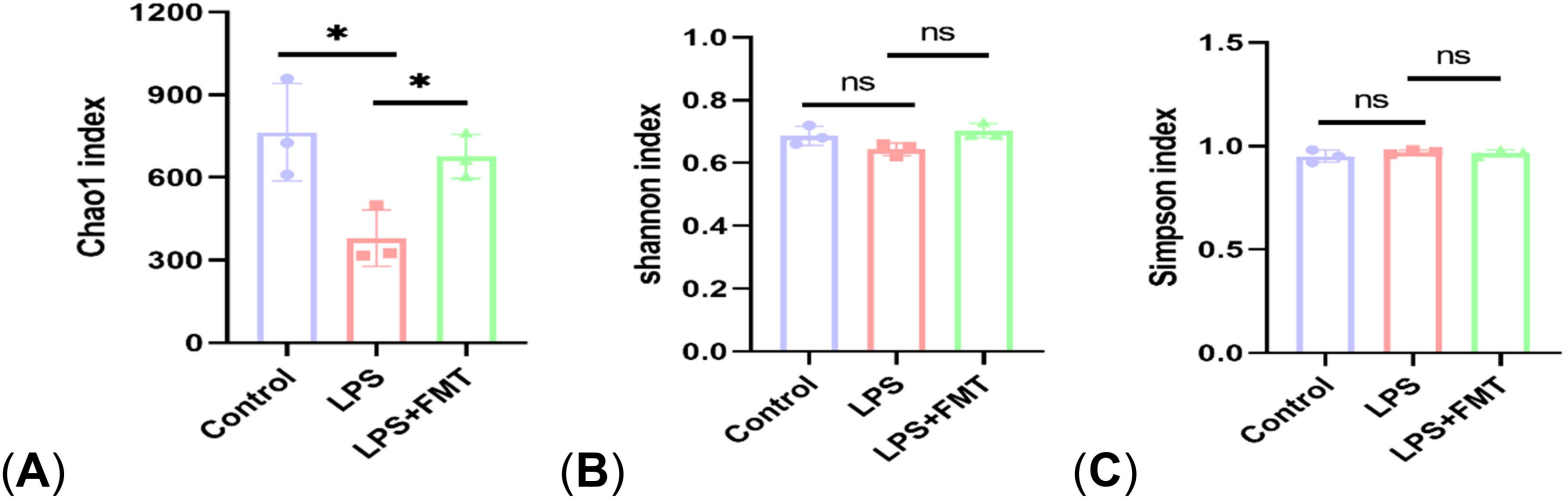
The α diversity analysis. **(A)** Chao 1 index; **(B)** Shannon index; **(C)** Simpson index. P<0.05 (*), P<0.01 (**), ns: no significance.

**Figure 5:**
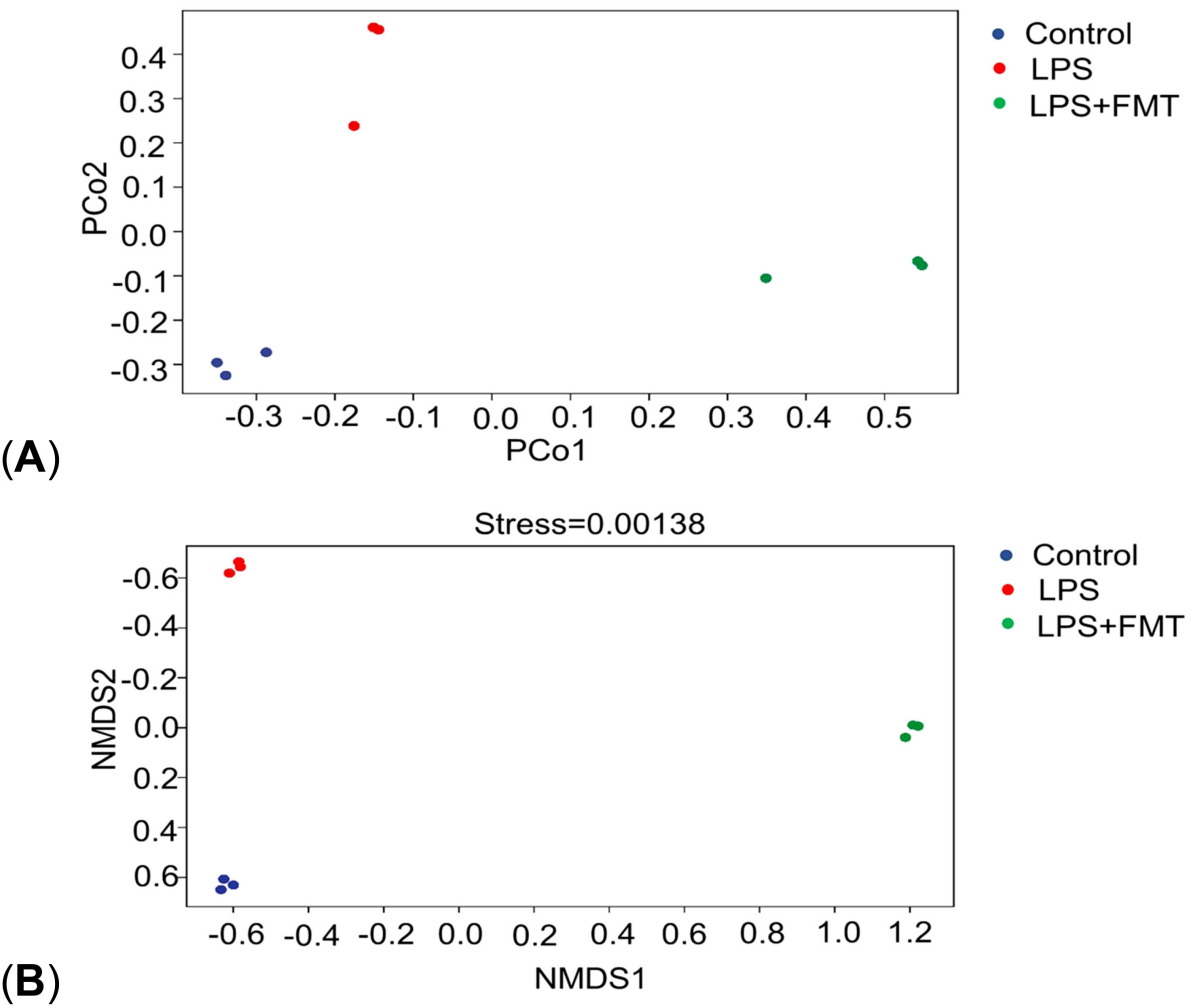
Results of PCoA and NMDS. **(A)** PCoA; **(B)** NMDS.

In terms of α diversity, there was a difference in the Chao1 index between the control and LPS groups (*P*<0.05), whereas the Chao1 index returned to normal levels after FMT treatment. It suggests that LPS can affect the abundance of gut microbiota in mice, causing the absolute number of gut microbiota to decrease, and FMT can eliminate this effect. There were no statistically significant differences in the Shannon and Simpson indices among the 3 groups, indicating that neither LPS nor FMT treatments had a significant impact on microbial diversity or the evenness of microbial distribution (**Figure 4**). In terms of β diversity, the results of both PCoA and NMDS showed that the same color points representing the samples within the group were close, that is, the composition of gut microbiota within the group was clustered with small differences. However, the different color points representing the different groups were far, indicating that the composition and ranking of gut microbiota between the groups were significantly different (**Figure 5**).

**Figure 6:**
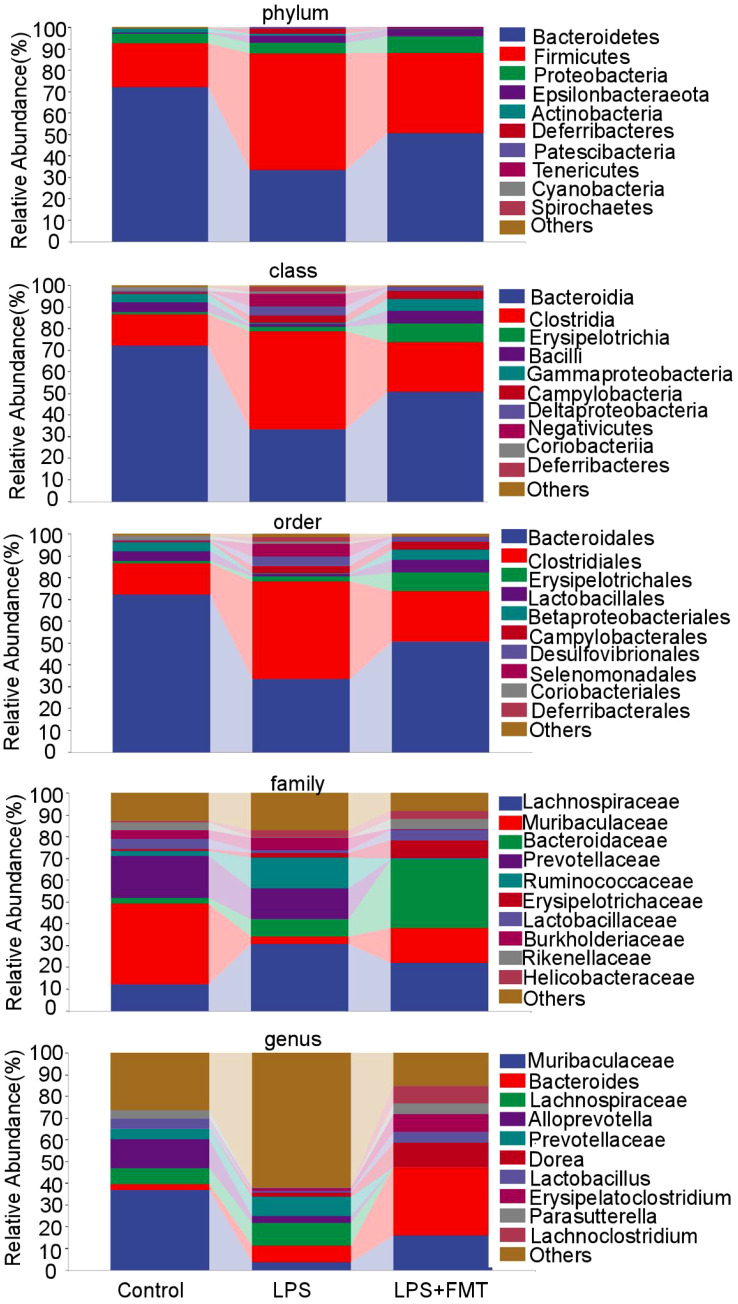
Analysis of gut microbiota composition at different taxonomic levels.

The relative abundance of the top 10 bacterial groups was analyzed. At the phylum level, the Bacteroidetes and Firmicutes were significantly different among the groups. At the class level, the abundance of Bacteroidia and Bacilli decreased in the LPS group. At the order level, the abundance of Muribaculaceae was reduced in the LPS group. All these changes returned to normal after the FMT treatment. At the family level, the abundances of Bacteroidaceae and Lactobacillaceae in the LPS+FMT group were higher than in the LPS group, while Ruminococcaceae showed higher abundance in the LPS group. Notably, at the genus level, the abundances of Bacteroides and Lactobacillus were significantly higher in the LPS+FMT group compared to the LPS group (**Figure 6**). These results suggest that FMT could modulate the balance of gut microbiota, such as increasing the levels of Bacteroides and Lactobacillus.

### 3.4 Comparative of immunofluorescence staining of femoral sections

**Figure 7:**
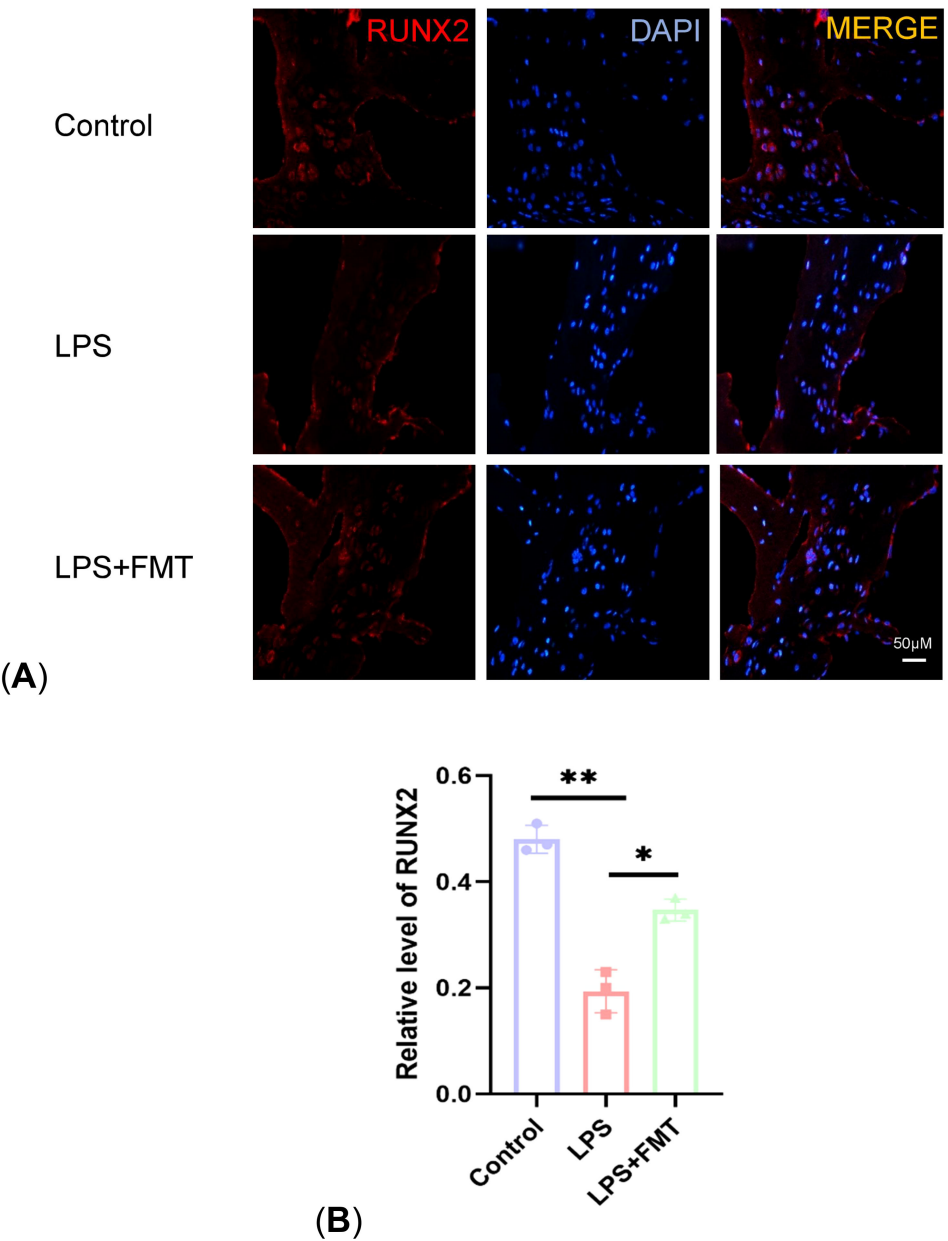
Comparison of RUNX2 expression levels. **(A)** Results of immunofluorescence staining; **(B)** Statistical plot of RUNX2 levels. P<0.05 (*), P<0.01 (**).

Osteoblasts are directly involved in bone formation and their number is negatively correlated with the severity of OP. In the study, immunohistochemical staining for its specific marker RUNX2 was performed. The results showed that the expression of RUNX2 in the LPS group was significantly lower than that in the control group (*P* < 0.01), while the expression of RUNX2 in the LPS + FMT group was significantly increased (**Figure 7**). These results indicated that FMT could alleviate LPS-induced OP by increasing the number of osteoblasts.

### 3.5 Determination of lncRNA *TUG1* levels in mouse gut and blood

**Figure 8:**
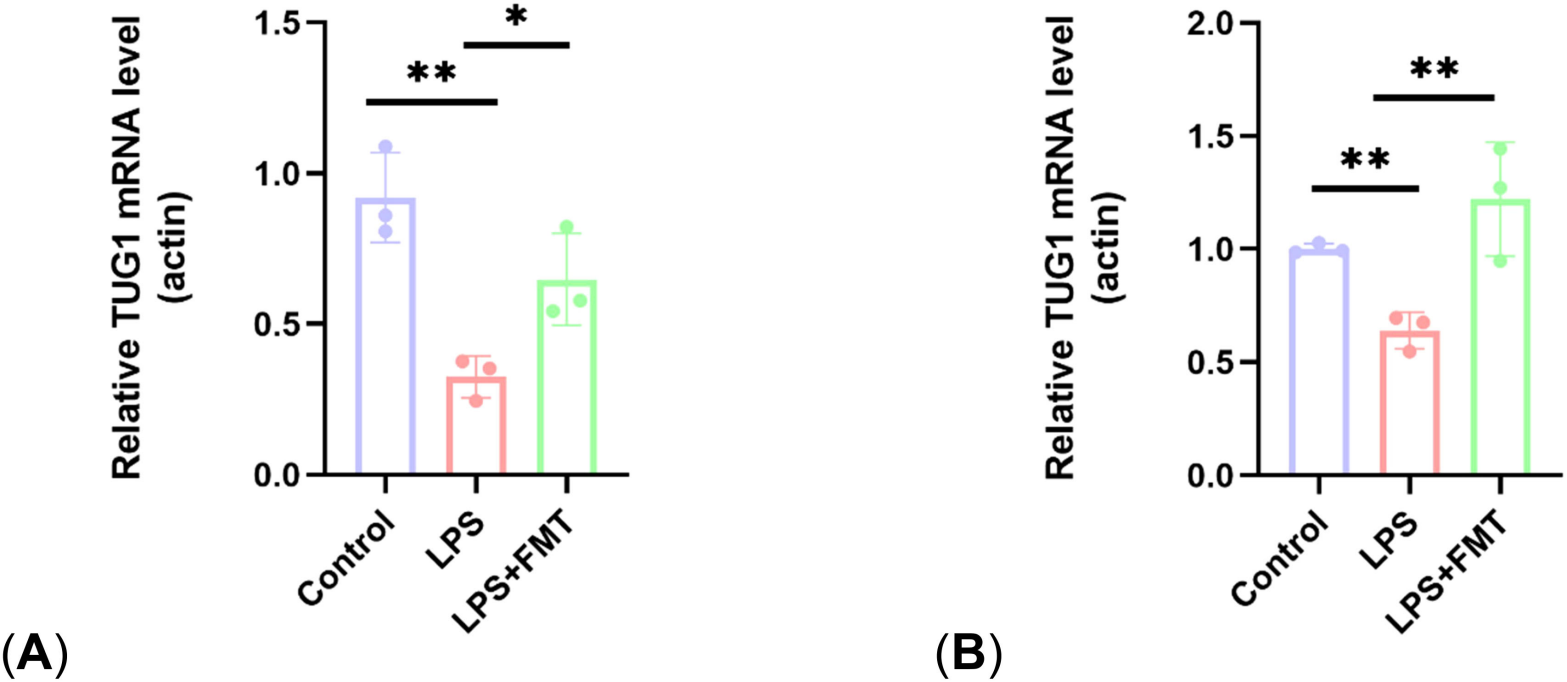
Comparison of lncRNA *TUG1* expression levels. **(A)** Expression levels of lncRNA *TUG1* in gut; **(B)** Expression levels of lncRNA *TUG1* in blood. P<0.05 (*), P<0.01 (**).

Bacteroides and Lactobacillus can regulate taurine production, and taurine can promote the expression of lncRNA *TUG1*, which regulates bone metabolism. To explore the mechanism by which FMT increases the number of osteoblasts, the expression levels of lncRNA *TUG1* in mouse gut and blood were examined in the study. The results showed that the *TUG1* mRNA levels in the LPS group were significantly lower than those in the control group, while the *TUG1* mRNA levels in the LPS + FMT group were significantly increased (**Figure 8**). These results suggest that FMT may alleviate OP by promoting the expression of lncRNA *TUG1* to restore the number of osteoblasts in OP mice.

## 4 Discussion

As the aging population intensifies, the number of OP patients is surging, imposing a significant economic burden on society (Gao et al., 2023). The results of this animal experiment indicate that FMT can enhance osteoblast levels and improve bone structure by modulating the abundance of gut microbiota in OP mice (such as increasing Bacteroides and Lactobacillus populations) and promoting the expression of lncRNA *TUG1*, thereby alleviating LPS-induced OP.

LPS is a common inducer of inflammatory OP. LPS activates specific pattern recognition receptors (PRRs) that detect microbial and infectious agents, triggering inflammatory responses, such as the LPS-TLR4/NF-κβ pathway, which produce inflammatory cytokines that impact the RANKL/RANK/OPG pathway leading to bone loss (Aquino-Martinez et al., 2020; de Vos et al., 2022). LPS also accelerates apoptosis in osteoblasts by increasing the expression of non-coding RNAs such as circular RNA circAtp9b and lncRNA TMC3-AS1 (Chen and Dai, 2022; Feng et al., 2022). Furthermore, LPS can promote oxidative stress damage by binding to the extracellular vesicles of harmful bacteria, compromising gut barrier functions (Chen S. et al., 2023). LPS-mediated inflammation also affects the gut microbiota, increasing the abundance of pathogenic bacteria such as Enterococcus spp. and Enterobacteriaceae (including Escherichia coli and Salmonella typhimurium). Oxidative stress inhibits microbiota protein synthesis, thereby reducing the metabolism of beneficial microbes to SCFAs and other substances (Kroon et al., 2025). The metabolic products of harmful bacteria enter the bloodstream through the compromised gut barrier, activating cellular immune responses, while the beneficial metabolic products like SCFAs, which are conducive to bone formation, decrease, ultimately leading to the onset of OP (Safdari et al., 2016; Candelli et al., 2021).

Traditional anti-OP medications often face issues like numerous side effects and prolonged treatment times, leading to poor patient compliance. Consequently, emerging biological therapies such as FMT are being considered to address these problems. The gut microbiota plays a crucial role in physiological and immune functions; any abnormal changes in its diversity and quantity can trigger adverse outcomes like bone metabolism disorders (Tyagi et al., 2018). FMT allows for the transfer of healthy donor gut microbiota into recipients to restore normal gut microecology. Compared to administering single probiotics, FMT has a longer-lasting effect on regulating gut microbiota and effectively controls the proliferation of harmful bacteria (Grehan et al., 2010). Currently, FMT has shown promising results in treating gastrointestinal diseases, but its efficacy and mechanisms in treating OP are still in preliminary exploration (Kim et al., 2024). This research findings indicate that FMT positively impacts alleviating LPS-induced OP. Previous studies have shown that after transplanting feces from juvenile rats, intestinal microbial structure improved in aged OP rats, with reduced bone loss (Ma et al., 2021). Another study demonstrated that FMT could enhance the expression of tight junction proteins between intestinal epithelial cells and inhibit the release of cytokines that promote osteoclastogenesis, thereby alleviating estrogen deficiency-induced OP (Zhang et al., 2022a). These pieces of evidence suggest that FMT has certain therapeutic effects in alleviating both primary and secondary OP.

The gut microbiota exhibits variations in species and abundance under different conditions such as age, diet, and disease. For instance, in the gut microbiota of aged OP rats, the abundance of the family Lachnospiraceae is higher, and this abundance decreases after the transplantation of feces from young rats (Ma et al., 2021). This study found that the abundance of Bacteroides and Lactobacillus in the gut microbiota of LPS-mediated OP mice was significantly increased after fecal transplantation from age-matched healthy mice. The gut microbiota can influence bone formation and resorption through its metabolic products or by regulating the host’s metabolism, immunity, and endocrine functions; this connection is referred to as the gut-bone axis. Different gut bacteria play distinct roles in this gut-bone axis, making it necessary to clarify the relationship between specific gut bacteria and bone metabolism. Research by Chen indicated that Lactobacillus rhamnosus can reduce the expression of pro-inflammatory cytokines and nuclear factor κB while increasing the expression of the anti-inflammatory cytokine interleukin-10, thereby improving bone quality (Chen YW. et al., 2023). Another study found that Akkermansia muciniphila can promote the synthesis of polyamines such as spermine and spermidine, which increase bone trabecular volume and density, thus enhancing the biomechanical strength of bones in adult female and young male mice (Chevalier et al., 2020). On the other hand, some gut bacteria can promote the onset of OP. A large sample study indicated that the abundance of Clostridiales bacterium DTU089 is negatively correlated with tibial bone density (Okoro et al., 2023). This study also found that the abundance of Ruminococcaceae is significantly higher in the LPS group than in other groups, possibly related to the LPS-mediated inflammatory response. Ruminococcaceae UCG004 has been shown to reduce bone mass and increase the risk of OP (Zeng et al., 2024). Clarifying the relationship between specific bacteria in the gut microbiota and OP can help deepen our understanding of their mechanisms and promote the development of biological agents for treating OP. The results of this study demonstrate that FMT can increase the numbers of Bacteroides and Lactobacillus. Additionally, after FMT, the abundance of Muribaculaceae is also restored, which can promote osteogenesis by producing SCFAs and regulating T-cell differentiation. These beneficial bacteria play a positive role in mitigating LPS-mediated OP.

LncRNAs are regulatory non-coding RNAs longer than 200 nucleotides that play a crucial role in the osteogenic differentiation of various stem cells. LncRNA *AC092155* can promote the osteogenic differentiation of adipose stem cells through the miR-*143*-*3p*/STMN1 axis (Sun et al., 2021). LncRNA *KCNMA1-AS1* can enhance the osteogenic differentiation of human bone marrow mesenchymal stem cells by activating the SMAD9 signaling pathway (Mai et al., 2023). Additionally, lncRNAs regulate certain bone metabolism markers and cytokines involved in bone metabolism. For instance, lncRNA *SNHG1* can inhibit the expression of osteoprotegerin by affecting its methylation status, thereby promoting bone resorption (Yu et al., 2022). LncRNA *CASC11* can drive the development of postmenopausal osteoporosis by upregulating tumor necrosis factor-α levels (Yu et al., 2019). Research has shown that Bacteroides and Lactobacillus can promote the methylation of lncRNA *MEG3* to inhibit osteoclastogenesis, indicating that gut microbiota can regulate bone metabolism by influencing lncRNA expression (Huang et al., 2022). In this study, FMT not only restored the abundance of Bacteroides and Lactobacillus but also elevated the levels of lncRNA *TUG1*. This may be related to certain metabolites produced by Bacteroides and Lactobacillus (such as taurine) that promote lncRNA *TUG1* expression. Bacteroides and Lactobacillus produce bile salt hydrolase (BSH), which hydrolyzes the C-24 N-acyl bond between taurine and bile acids, breaking down taurocholate-a compound of bile acids and taurine entering the intestine via bile-into free bile acids and taurine (Cai et al., 2022). Taurine, a cysteine derivative, can bind to glycine-receptor α2 and GABA(A) receptors, releasing signals that upregulate the level of lncRNA *TUG1* (Young et al., 2005). LncRNA *TUG1* can facilitate the differentiation of bone marrow mesenchymal stem cells into osteoblasts and inhibit their apoptosis by targeting the miRNA-*34a*/FGFR1 pathway (Wang et al., 2021a). These studies illustrate the potential for Bacteroides and Lactobacillus to increase bodily taurine levels and subsequently upregulate lncRNA *TUG1*. These theories may support the results of our study, which showed that fecal transplantation could alleviate LPS-mediated OP by regulating the expression of lncRNA *TUG1*. The study also found that the restoration levels of lncRNA *TUG1* in the blood post-FMT were higher than in the gut, which may be related to the different ability of lncRNA *TUG1* expression in different tissue cells. A study found that *TUG1* levels are higher in the central nervous system than in other tissues (Subhramanyam and Hu, 2017). Another study found that *TUG1* was mainly distributed in the cytoplasm and nucleus, so the distribution of *TUG1* in different cellular structures was different (Liu et al., 2020). However, the reasons for these differences still need further investigation.

Research indicates that Lactobacillus can drive RNA m6A modification mediated by METTL3 through its production of lactic acid, thereby upregulating lncRNA *TUG1* (Xiong et al., 2022; Xi et al., 2024). Bacteroides can downregulate the TLR4/NF-κβ signaling pathway through its production of capsular polysaccharide A, reversing LPS-mediated inflammatory responses (Wang et al., 2021b). These findings reveal potential mechanisms by which Lactobacillus and Bacteroides influence LPS-mediated OP through other metabolic products, with more mechanisms yet to be explored.

In addition to affecting the gut microbiota and levels of lncRNA *TUG1*, FMT can influence bone metabolism by regulating gut barrier functions and the levels of inflammatory factors. FMT increases the expression of ZO-1 and Occludin to restore gut barrier function and inhibits the release of TNF-α and IL-1 β to suppress osteoclast formation, all of which are beneficial for alleviating OP (Zhang et al., 2022a). Moreover, transplanting feces from elderly OP rats to young rats compromised the intestinal barrier of the young (Wang N. et al., 2022). Transplanting feces from postmenopausal OP women to germ-free mice treated with antibiotics elevated levels of TNF-α and IL-17A in the mice (Chen et al., 2024). These studies highlight the roles of gut barrier integrity and systemic inflammatory status in FMT.

Previous animal studies on FMT for the treatment of OP mostly used elderly animal models or animal models with estrogen deficiency after ovariectomy. In this study, LPS was used to establish animal models of inflammation-mediated OP, and the positive role of FMT in the treatment of LPS-mediated OP and the possible mechanism were investigated. This study also found that the number of Bacteroides and Lactobacillus in the intestine of OP mice increased after FMT treatment, and identifying these potentially beneficial bacteria can provide reference for other treatment methods such as intake of probiotics. This study is the first to focus on the changes in the level of LncRNA *TUG1* during FMT treatment of OP, and lncRNA *TUG1* has been shown to promote the proliferation of osteoblasts and inhibit their apoptosis. The results of this study can provide new ideas for the mechanism of FMT treatment of OP.

However, there are certain limitations in this study. The study focused solely on the changes in the gut microbiota and lncRNA *TUG1* levels following FMT, but did not evaluate the effects of changes in gut barrier function and inflammatory factor levels on bone metabolism. Furthermore, it did not analyze the functional aspects of the gut microbiota through metabolomics. This study noted an increase in the abundance of Bacteroides and Lactobacillus post-FMT and highlighted the elevated levels of lncRNA *TUG1*. Building on previous studies on taurine metabolism and efficacy, it outlined a positive pathway involving Bacteroides/Lactobacillus-taurine-lncRNA *TUG1* (Young et al., 2005; Cai et al., 2022). Future studies will consider incorporating Phylogenetic Investigation of Communities by Reconstruction of Unobserved States or metabolomics analyses to provide more comprehensive evidence for the mechanisms by which FMT alleviates OP. Additionally, the functional assays for lncRNA *TUG1* need to be improved, such as clarifying whether knocking down lncRNA *TUG1* can reverse the effects of FMT. These issues will be addressed in further research.

Currently, FMT faces numerous challenges in treating human OP. Inappropriate donor selection can not only fail to treat OP but also increase the presence of harmful components in the gut microbiota or even lead to cross-infection. Donor screening is difficult and costly; one study showed that only about 10% of potential donors passed the screening due to reasons such as medication use, parasitic infections, and Helicobacter pylori infections, with the entire process costing 64.112 euros (Bénard et al., 2022). The selection of recipients is equally important; causes of OP include aging, inflammation, and estrogen deficiency, and it is unclear which subtypes benefit most from FMT. It is also uncertain whether FMT could cause adverse reactions in patients with other diseases or those taking other medications (Zhang et al., 2022b). Future research should seek solutions to these issues, further standardize the selection of fecal donors and recipients, and the transplantation routes, explore the appropriate number of transplantations, and weigh the pros and cons of combination therapies. For treating different subtypes of OP, personalized microbiome intervention strategies should be a direction for future research, such as personalized enrichment treatments with beneficial gut bacteria. Additionally, establishing a large-scale microbial library based on standard healthy donor gut microbiota could help reduce the risks and costs associated with FMT. As research continues to deepen, it is anticipated that fecal transplantation therapy for OP will bring more benefits to patients and society.

## Data Availability

The original contributions presented in the study are included in the article/supplementary material. Further inquiries can be directed to the corresponding authors.
